# Large cyst of the vaginal wall in pregnancy

**DOI:** 10.1002/ccr3.2370

**Published:** 2019-08-08

**Authors:** Ioannis K. Papapanagiotou, Ioannis Chatzipapas, Pelopidas Koutroumanis, Spyridon Marinopoulos, Nikolaos Thomakos, Maria Brozou, Konstantinos Ntzeros, Evangelos Petrakis, Michalis Diakosavvas, Zacharias Fasoulakis, George Morphopoulos, Dimitrios Loutradis

**Affiliations:** ^1^ 1st Department of Obstetrics and Gynecology General Hospital “Alexandra” Athens Greece; ^2^ Department of Pathology General Hospital “Alexandra” Athens Greece

**Keywords:** gestation, protruding membranes, vaginal cyst, vaginal wall

## Abstract

Large vaginal cysts during pregnancy are rare and can mislead Obstetricians to a false diagnosis, that of “Protruding membranes”. Aspiration of the cyst can be easily performed, resulting in the collapsing of the cyst and an uneventful vaginal delivery can be conducted.

## 1 QUESTION

What is this condition and how it should be treated?

## 2 ANSWER

**Figure 1:**
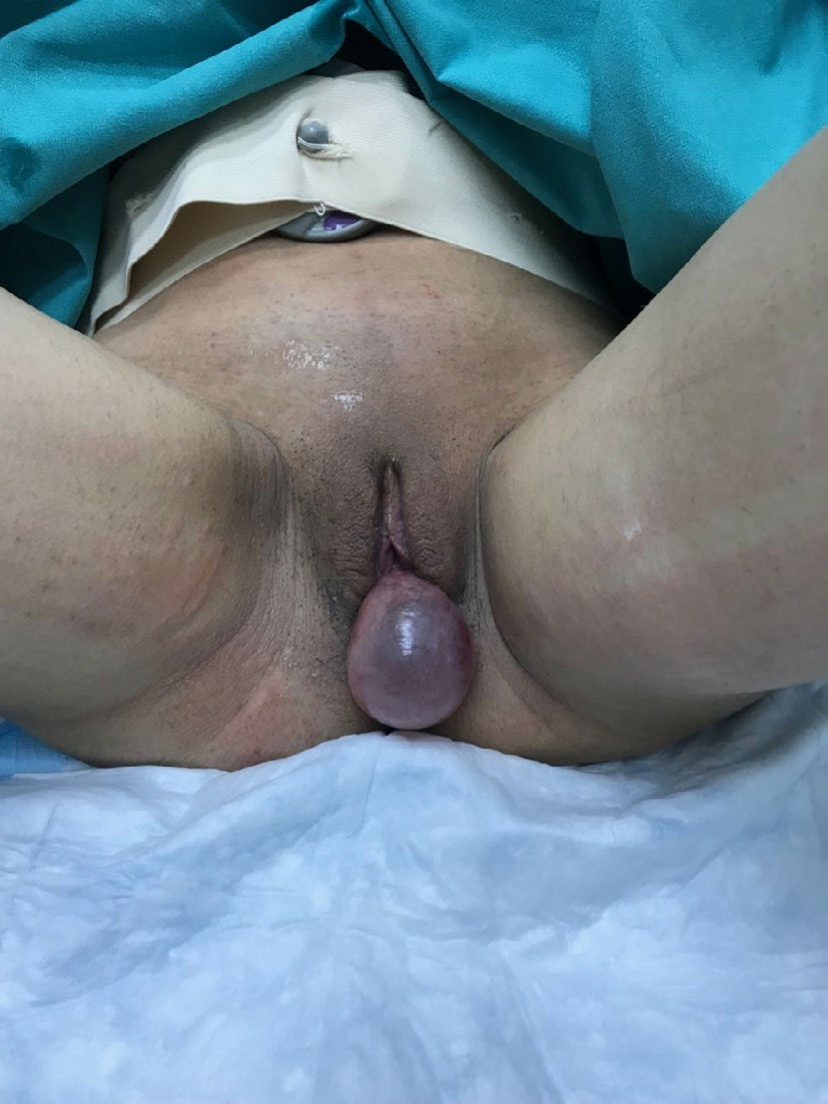
A large tense vaginal cyst during pregnancy measuring 5 × 6 cm

**Figure 2:**
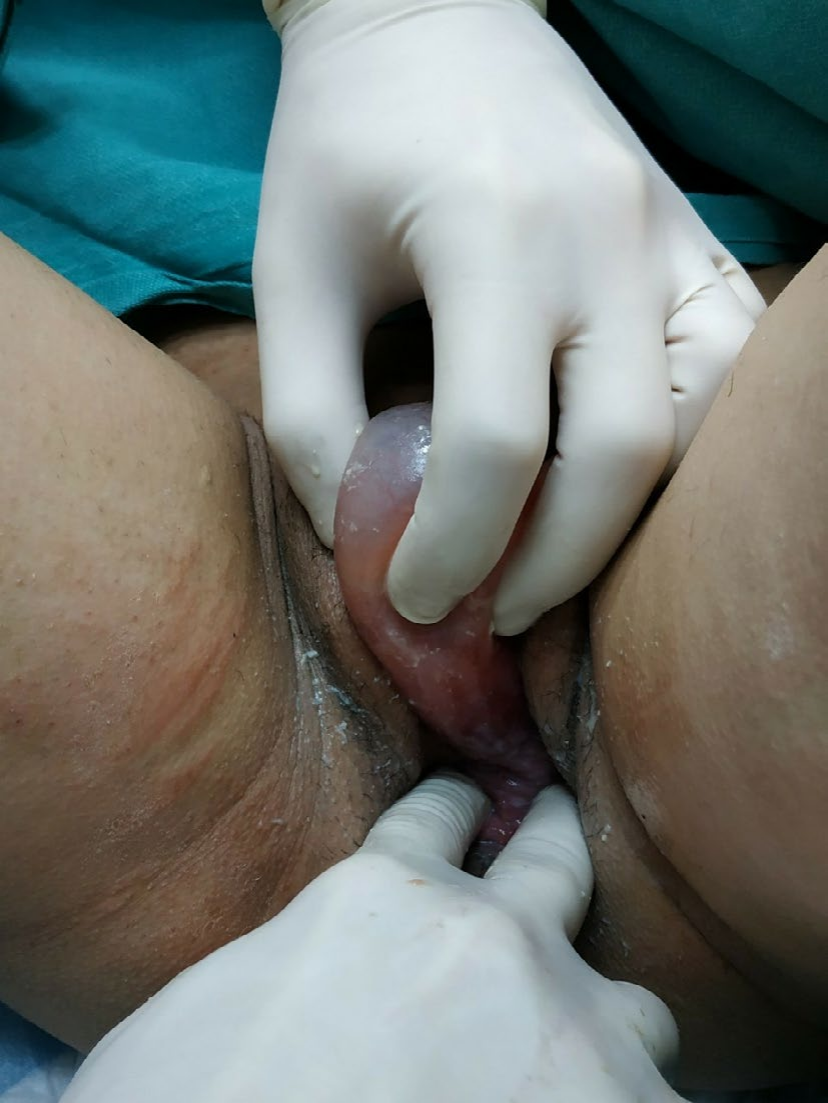
Clinical examination revealed the origin of the large tense cyst from the upper left lateral wall of her vagina

**Figure 3:**
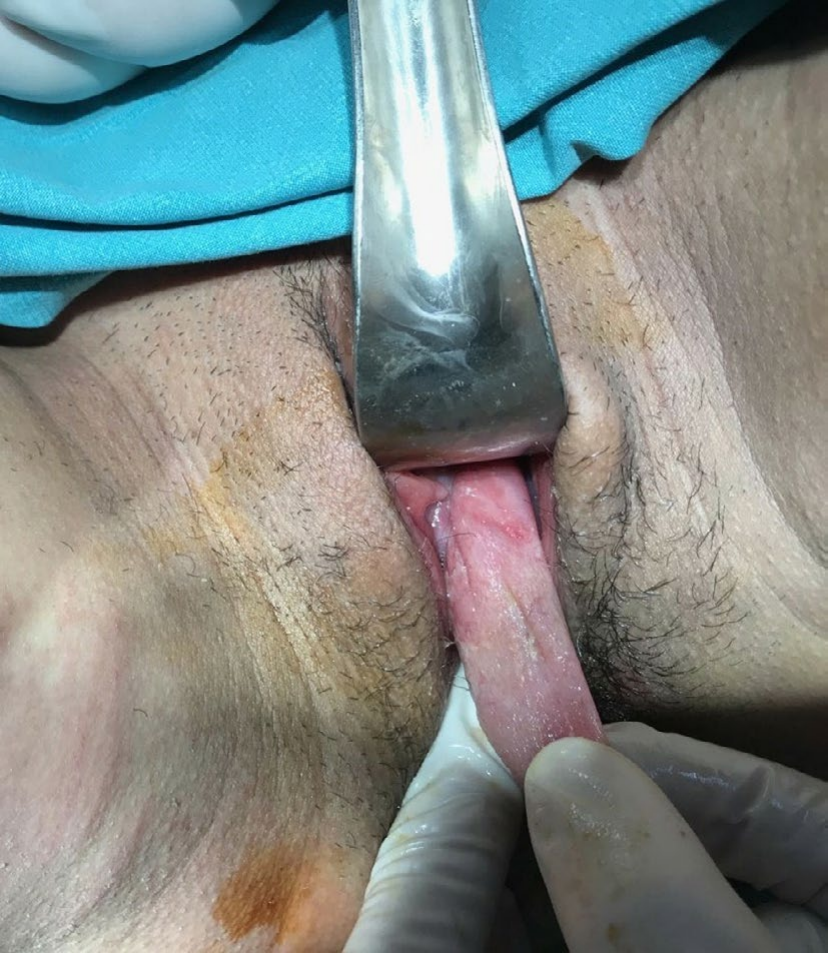
Aspiration of the cyst was performed, resulting in the collapsing of the cyst

**Figure 4:**
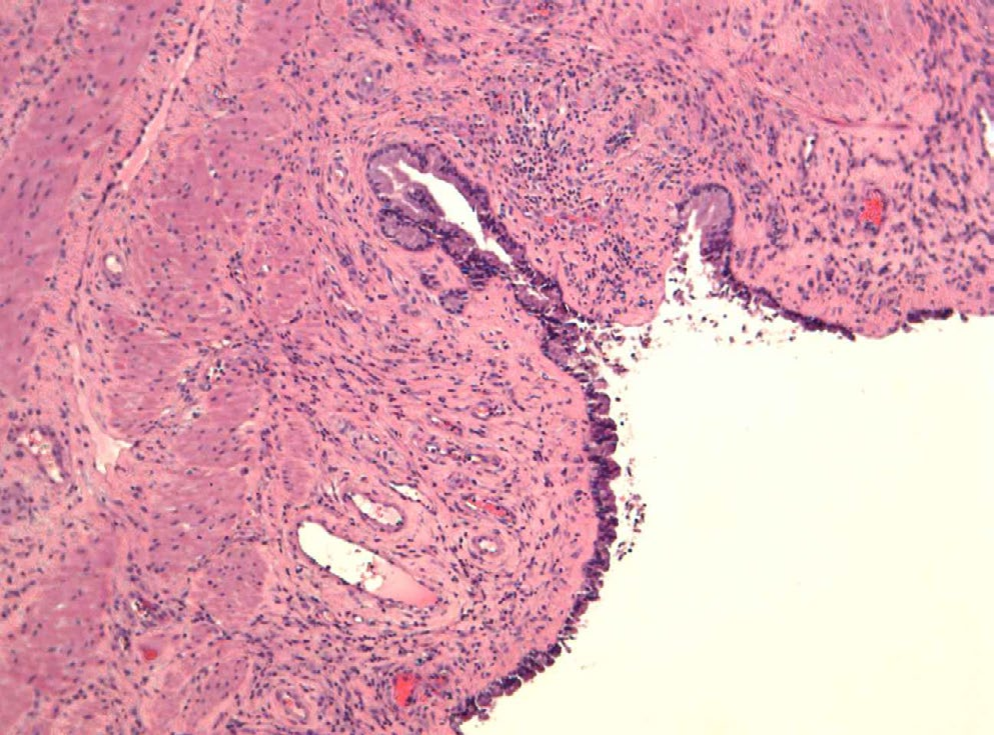
Müllerian cyst with squamous metaplasia (H&E stain)

A 23‐year‐old pregnant woman (para 1, gravida 2) going through her 39th week of gestation was referred to our Emergency Obstetrics Department with a diagnosis of “protruding membranes” (Figure 1). Clinical examination revealed a protruding mass, a large tense cyst measuring 5 × 6 cm arising from the upper left lateral wall of her vagina (Figures 2 and 3). Subsequently, aspiration of the cyst was performed, resulting in the collapsing of the cyst, and an uneventful vaginal delivery was conducted. Following delivery, the cyst was excised and vaginal wall repaired. FNA did not reveal the presence of malignant tissue. On histopathology, the cyst was identified as a Müllerian cyst (Figure 4). The patient recovered and remained asymptomatic on follow‐up.

According to the literature, this is one of the few incidences reported,^1^, ^2^ in which this large tense cyst occurs during pregnancy and can misguide and stress physicians setting the diagnosis of “protruding membranes”. Impressively, this cyst was formed, as described by the patient, during the last 1 week. On the other hand, in a previously reported case, the cyst was present since puberty but increased in size during pregnancy.^1^

## CONFLICT OF INTEREST

None declared.

## AUTHOR CONTRIBUTIONS

IKP: handled the patient in the Emergency Room, conceived and wrote the clinical case. IC: treated the patient, provided critical revision of the article. PK: treated the patient, provided critical revision of the article. SM: treated the patient. NT: collected the data. MB: collected the data. KN: was responsible for patient's labor. EP: was responsible for patient's labor. MD: co‐wrote the clinical case. ZF: handled the patient in the Emergency room. GM: performed histological analysis of the vaginal cyst. DL: provided final approval of the version to publish.
